# Semaglutide and Liraglutide Modulate Oxidative Stress and Wound Repair in Normal Human Skin Cells Exposed to an In Vitro Diabetic-like Environment

**DOI:** 10.3390/ijms27156981

**Published:** 2026-08-03

**Authors:** Ioanna A. Anastasiou, Konstantinos Tentolouris, Athanasia Katsaouni, Panagiotis Sarantis, Iordanis Mourouzis, Eleni Rebelos, Nikolaos Tentolouris

**Affiliations:** 1 1st Department of Propaedeutic Internal Medicine, Medical School, National and Kapodistrian University of Athens, ‘Laiko’ General Hospital, 11527 Athens, Greece; elenirebelos@gmail.com; 2 Department of Pharmacology, Medical School, National and Kapodistrian University of Athens, 11527 Athens, Greece; kostasten@med.uoa.gr (K.T.); athanasia.paraskevikatsaouni@gmail.com (A.K.); imour@med.uoa.gr (I.M.); 3 Molecular Oncology Unit, Department of Biological Chemistry, Medical School, National and Kapodistrian University of Athens, 11527 Athens, Greece; panayotissarantis@gmail.com; 4 Department of Clinical and Experimental Medicine, University of Pisa, 56126 Pisa, Italy

**Keywords:** semaglutide, liraglutide, oxidative stress, wound healing, cellular regeneration, diabetic complications

## Abstract

Glucagon-like peptide-1 receptor agonists (GLP-1 RAs) not only improve glycemic control but also possess anti-inflammatory and antioxidant properties. In this study, the effects of semaglutide and liraglutide on oxidative stress and wound healing were examined in human dermal fibroblasts (NHDFs) and keratinocytes (NHEKs) under diabetes-mimicking conditions. Cells were exposed to high glucose and hydrogen peroxide, followed by treatment with semaglutide or liraglutide at concentrations of 22.5 and 45 pg/mL for 48 h. We assessed cell viability, apoptosis, intracellular reactive oxygen species (ROS), Ki-67, and Pax-7. Advanced glycation end-products (AGEs) and their receptor (RAGE) were quantified via ELISA. Wound healing was evaluated by scratch assay, and the gene expression of antioxidants, cytokines, and matrix components was measured by real-time PCR. Semaglutide significantly improved NHDF viability and proliferation under diabetic conditions, reducing ROS production more effectively than liraglutide. Apoptosis decreased, evidenced by increased Bcl-2 alongside decreased Bax and cleaved caspase-3, particularly with semaglutide. Furthermore, semaglutide uniquely activated Pax-7 expression. While both agents upregulated antioxidants, semaglutide more effectively suppressed AGEs/RAGE and inflammatory cytokines. Both drugs enhanced collagen expression and accelerated wound closure; however, semaglutide achieved near-complete healing within 48 h. Neither drug induced observable changes in NHEKs. Semaglutide exhibits strong antioxidative, cytoprotective, and pro-regenerative effects in diabetic NHDFs, outperforming liraglutide across multiple parameters. These findings highlight semaglutide’s therapeutic potential for diabetic wound healing, warranting further in vivo investigation.

## 1. Introduction

Diabetes mellitus (DM) is a global health problem that affects more than 463 million adults around the world [1]. Studies show that the number of people with the disease will continue to rise in the coming decades [2]. Chronic hyperglycemia in diabetes mellitus (DM) leads to both microvascular and macrovascular complications, including impaired wound healing that frequently progresses to non-healing diabetic foot ulcers (DFUs) and lower-limb amputations [3,4]. Multiple factors contribute to defective wound repair in individuals with DM, such as persistent inflammation, excessive oxidative stress, dysregulated extracellular matrix remodeling, and reduced cellular proliferation and migration [5,6].

Glucagon-like peptide-1 (GLP-1), an incretin hormone secreted by intestinal L-cells in response to nutrient intake, exerts glucose-dependent insulinotropic effects and plays a key role in maintaining glucose homeostasis [7]. GLP-1 is thought to control many different physiological processes in many different organs, in addition to its metabolic effects. For example, it can change inflammatory pathways and redox homeostasis [8,9]. Glucagon-like peptide-1 receptor agonists (GLP-1 RAs), such as semaglutide and liraglutide, are widely used in individuals with type 2 diabetes (T2D) because they provide robust glycemic control and have well-established cardiovascular safety profiles [10]. Recent in vitro, in vivo and clinical studies have suggested that GLP-1 RAs may have multiple effects, including anti-inflammatory and antioxidant effects, that are not related to their effects on blood sugar levels. These effects could be useful for healing wounds and repairing tissue [11,12]. Human dermal fibroblasts and keratinocytes play essential roles in wound healing, acting in coordination to regulate cell proliferation, migration, extracellular matrix synthesis, and cytokine secretion [13]. Importantly, epidermal and dermal cells express the GLP-1 receptor and thus GLP-1 RA may have direct actions on the cells, an issue that would be particularly important in terms of wound healing in diabetic ulcers [14].

In this study, we compared the effects of semaglutide and liraglutide versus a control on oxidative stress, apoptosis, and wound-healing responses in human dermal fibroblasts and keratinocytes cultured under diabetes-mimicking conditions.

## 2. Results

### 2.1. Toxicity/Viability Assessment and Cell Death Assessment

**Figure 1:**
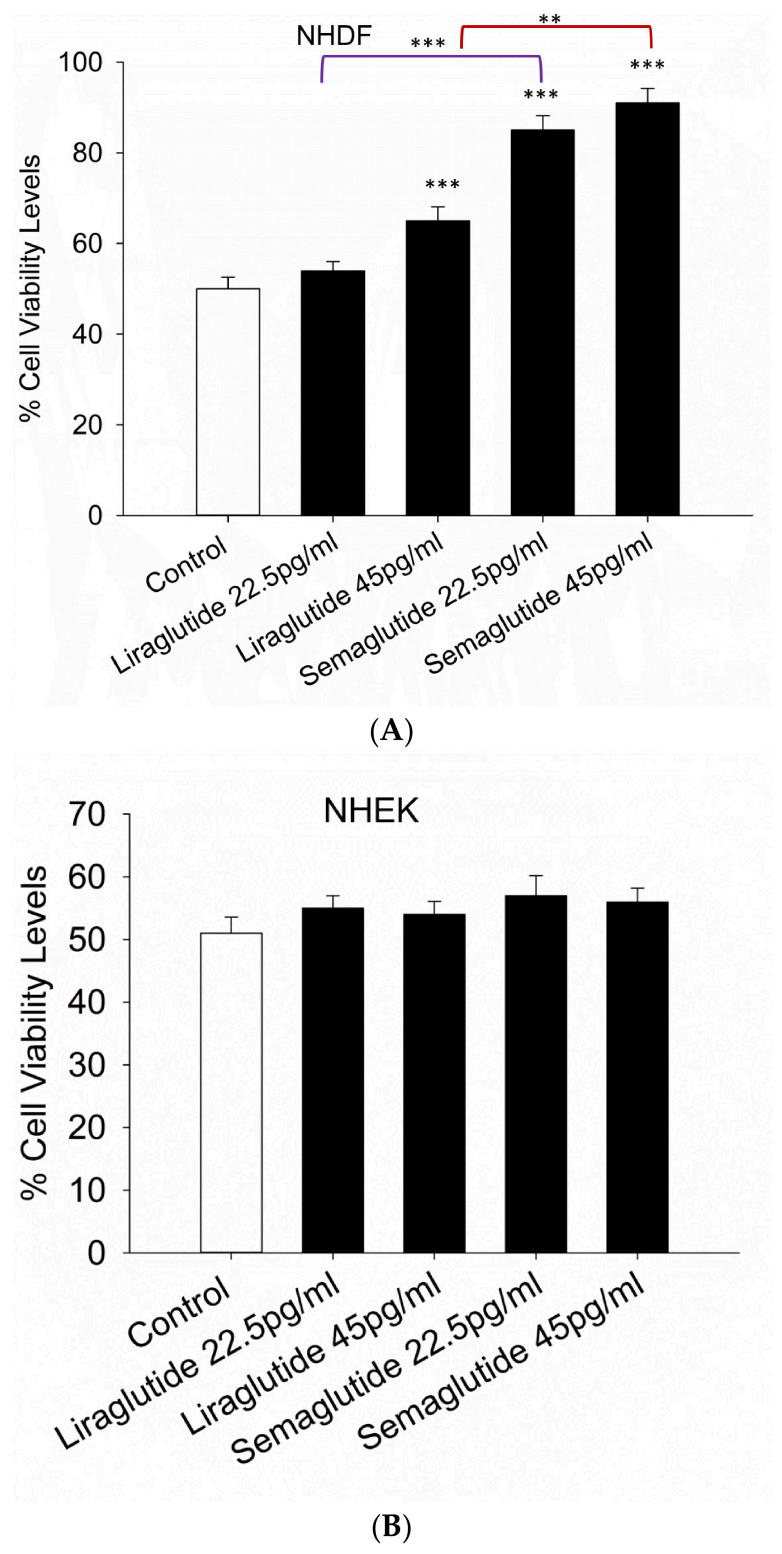
Assessment of cell viability. (**A**,**B**). Cell viability in NHDF and NHEK cells was evaluated using the MTT assay. Cells were exposed to a diabetic-like environment and treated with liraglutide or semaglutide at concentrations of 22.5 and 45 pg/mL for 48 h. Results are presented as mean ± SD from three independent experiments (N = 6). Statistical analysis was performed using one-way ANOVA followed by Dunnett’s T3 post hoc test. Significance is indicated as *** *p* < 0.001 versus the control group. Comparisons between the two drug treatment groups were performed using an independent-samples *t*-test, with significance indicated as ** *p* < 0.01 and *** *p* < 0.001. ANOVA, analysis of variance; MTT, 3-(4,5-dimethylthiazol-2-yl)-2,5-diphenyltetrazolium bromide; NHDF, normal human dermal fibroblasts; NHEK: normal human epidermal keratinocytes; SD, standard deviation.

To assess the effects of liraglutide and semaglutide on cell viability, NHDF and NHEK cells were exposed to a diabetic environment and treated with liraglutide or semaglutide at two concentrations (22.5 and 45 pg/mL) for 48 h, then analyzed by MTT assay. In NHDFs, liraglutide at 45 pg/mL and semaglutide at all tested concentrations significantly enhanced cell viability compared with the control group (*p* < 0.001). Moreover, at 22.5 and 45 pg/mL, semaglutide produced a greater increase in cell viability than liraglutide at the corresponding concentrations, as shown by inter-drug comparisons. In contrast, in NHEK cells, neither liraglutide nor semaglutide affected cell viability under diabetic conditions (Figure 1A,B). Supplementary Figure S2 shows that semaglutide and liraglutide exert no detectable effects on viability of normal NHDF and NHEK cells under non-diabetic conditions, indicating that their actions are context-dependent and become apparent only in a diabetic or stress-induced environment.

### 2.2. Apoptosis Levels

**Figure 2:**
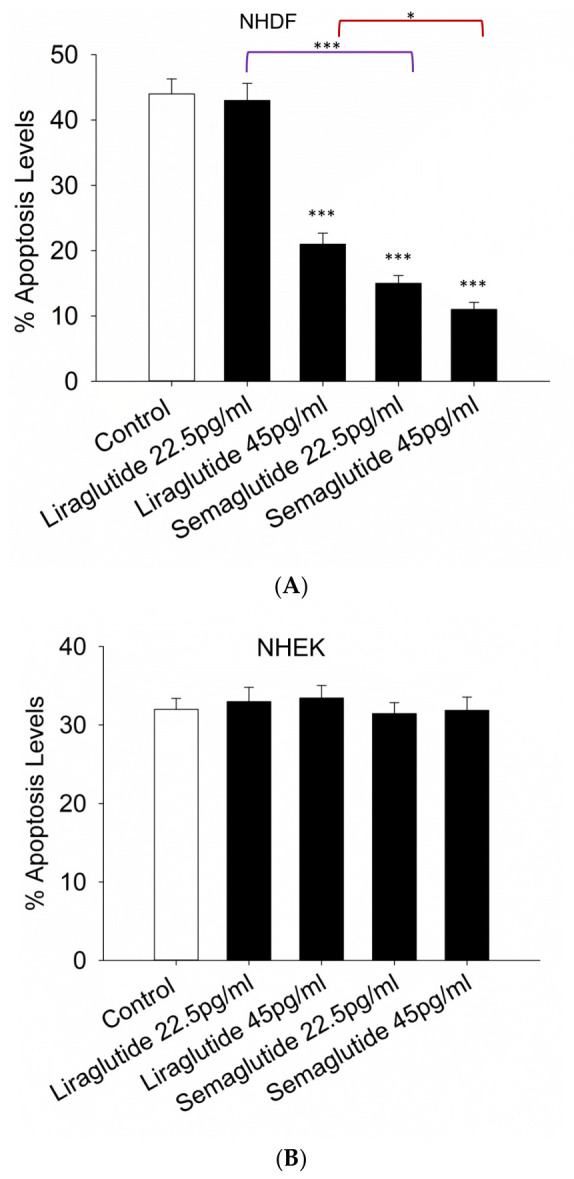
Assessment of apoptosis levels. (**A**,**B**). Bar graph showing apoptosis levels in NHDF and NHEK cells under diabetic conditions after 48 h of treatment with liraglutide or semaglutide (22.5 and 45 pg/mL). Apoptosis was assessed by flow cytometry using propidium iodide (PI) staining. Data are presented as mean ± SD from three independent experiments (N = 3). Statistical significance was determined using one-way ANOVA followed by Dunnett’s T3 post hoc test. Significance levels are indicated as *** *p* < 0.001, compared to the control group. Comparisons between the two drug treatment groups were performed using an independent-samples *t*-test, with significance indicated as * *p* < 0.05 and *** *p* < 0.001. ANOVA, analysis of variance; NHDF, normal human dermal fibroblasts; NHEK: normal human epidermal keratinocytes; SD, standard deviation.

Apoptosis in NHDF and NHEK cells was assessed after exposure to a diabetic environment and subsequent treatment with liraglutide or semaglutide. In NHDFs, liraglutide at 45 pg/mL and semaglutide at 22.5 and 45 pg/mL significantly reduced apoptosis compared with the control. Notably, semaglutide at 22.5 and 45 pg/mL exerted stronger anti-apoptotic effects than liraglutide at the corresponding concentrations in inter-drug comparisons. In contrast, apoptosis levels in NHEK cells remained unchanged across all treatment conditions (Figure 2A,B).

### 2.3. Immunofluorescence of Ki-67 and Pax-7 Positive Cells

**Figure 3:**
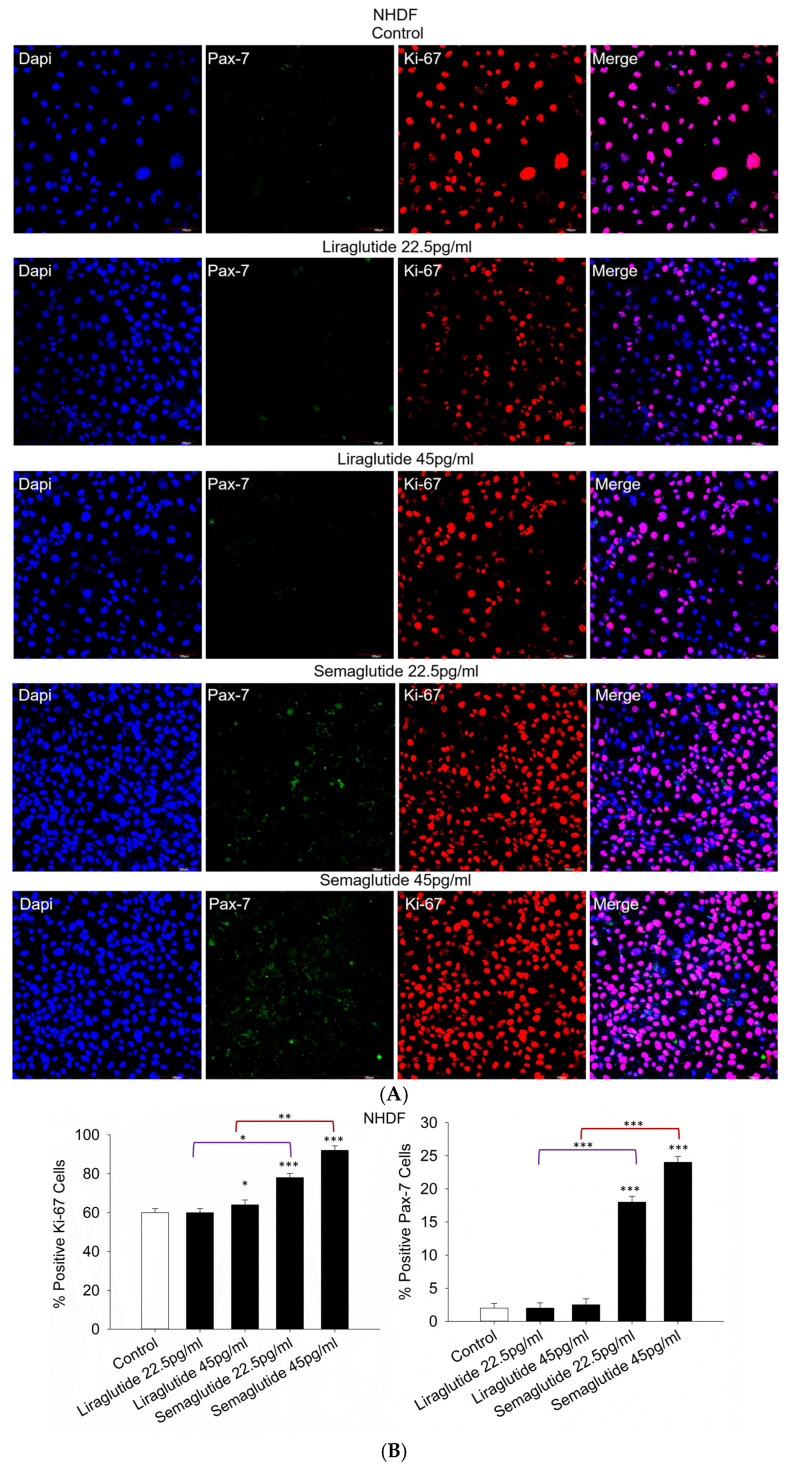

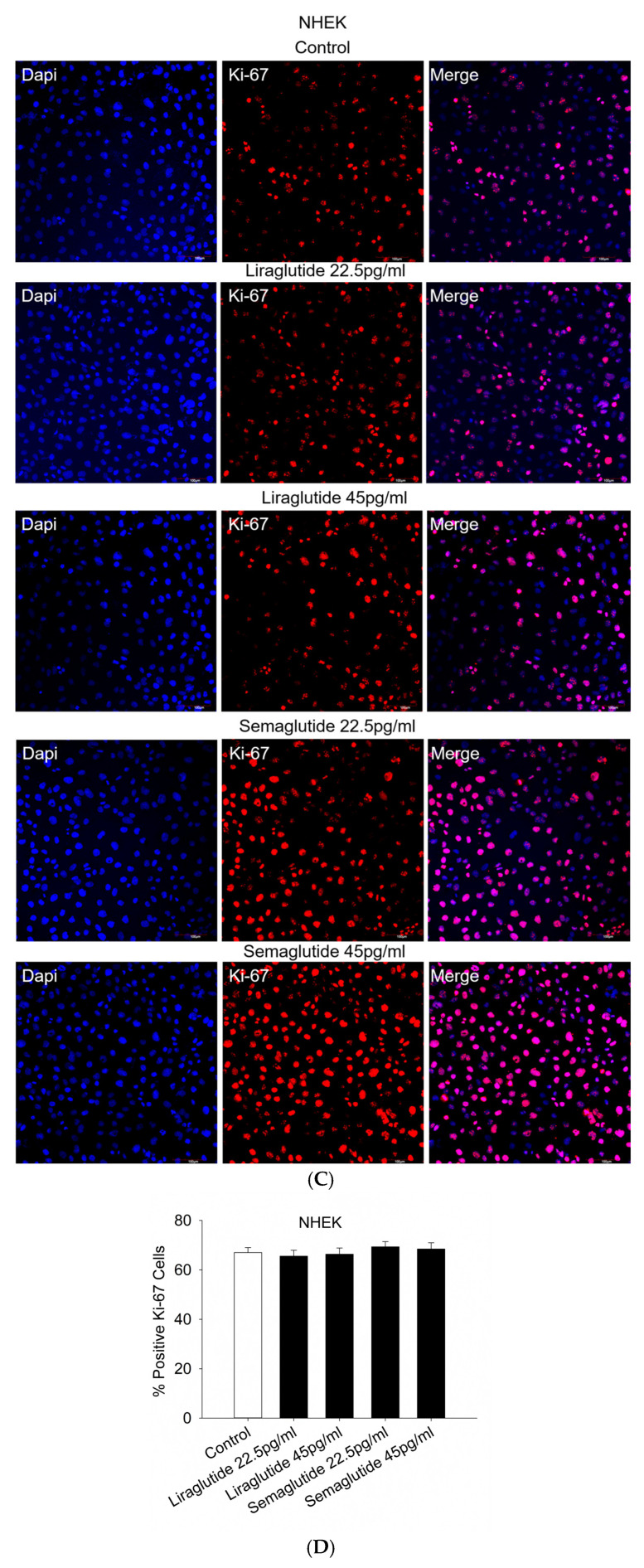
Evaluation of cell proliferation in NHDF and NHEK and Pax-7 expression in NHDF. (**A**). NHDF cells were exposed to a diabetic environment and treated with liraglutide or semaglutide (22.5 or 45 pg/mL) for 48 h. The first column shows nuclear DAPI staining (blue), the second Pax-7 (green), the third Ki-67 (red), and the fourth the merged images of Pax-7, Ki-67 and DAPI. Images were acquired at 20× magnification; scale bar = 100 μm. (**B**). Quantitative analysis of Pax-7 and Ki-67 fluorescence in NHDFs, expressed as mean ± SD from three independent experiments (N = 3). Statistical significance was evaluated by one-way ANOVA followed by Dunnett’s T3 post hoc test; * *p* < 0.05, *** *p* < 0.001 versus control. Comparisons between the two drug treatment groups were performed using an independent-samples *t*-test, with significance indicated as * *p* < 0.05, ** *p* < 0.01 and *** *p* < 0.001. (**C**). NHEK cells were exposed to a diabetic environment and treated with liraglutide or semaglutide (22.5 or 45 pg/mL) for 48 h. The first column shows nuclear DAPI staining (blue), the second Ki-67 (red), and the third the merged Ki-67/DAPI images. Images were acquired at 20× magnification; scale bar = 100 μm. (**D**). Quantification of Ki-67 fluorescence in NHEKs, shown as mean ± SD from three independent experiments (N = 3). Statistical analysis was performed using one-way ANOVA followed by Dunnett’s T3 post hoc test. ANOVA, analysis of variance; DAPI, 4′,6-diamidino-2-phenylindole; NHDF, normal human dermal fibroblasts; NHEK, normal human epidermal keratinocytes; Pax-7, Paired Box 7; SD, standard deviation.

Semaglutide induced a dose-dependent, significant increase in NHDF proliferation by Ki-67 immunofluorescence. Liraglutide produced a modest, significant increase only at the higher dose, while the lower liraglutide dose did not differ from control. Pax-7 expression, assessed only in NHDF cells as a marker of Pax-7 positive cells, was markedly upregulated by semaglutide at both doses; liraglutide had no significant effect on Pax-7. Notably, semaglutide at 22.5 and 45 pg/mL induced higher proliferation and Pax-7 expression than liraglutide at the corresponding concentrations in inter-drug comparisons. No significant changes in Ki-67 were observed in NHEK cells under any treatment condition (Figure 3A–D).

### 2.4. Intracellular ROS Production and Mitochondrial Superoxide Production

**Figure 4:**
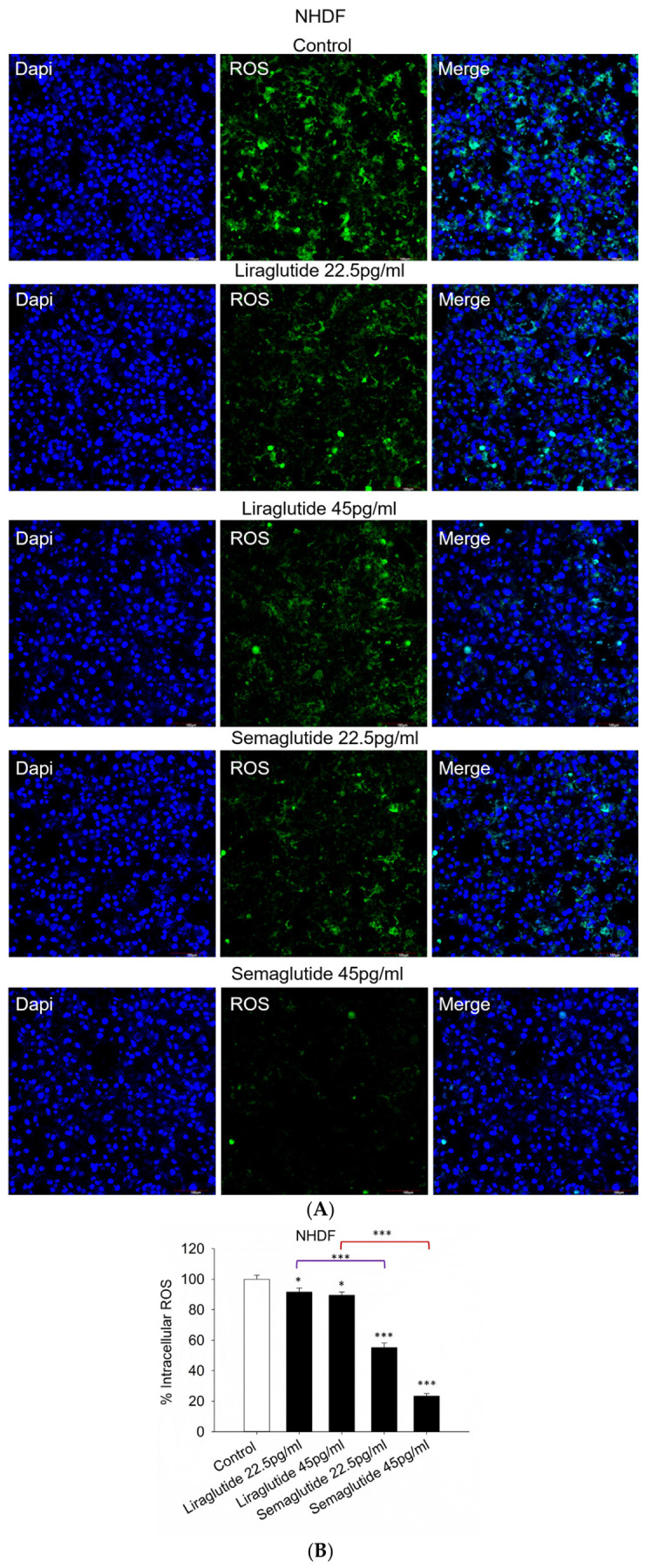

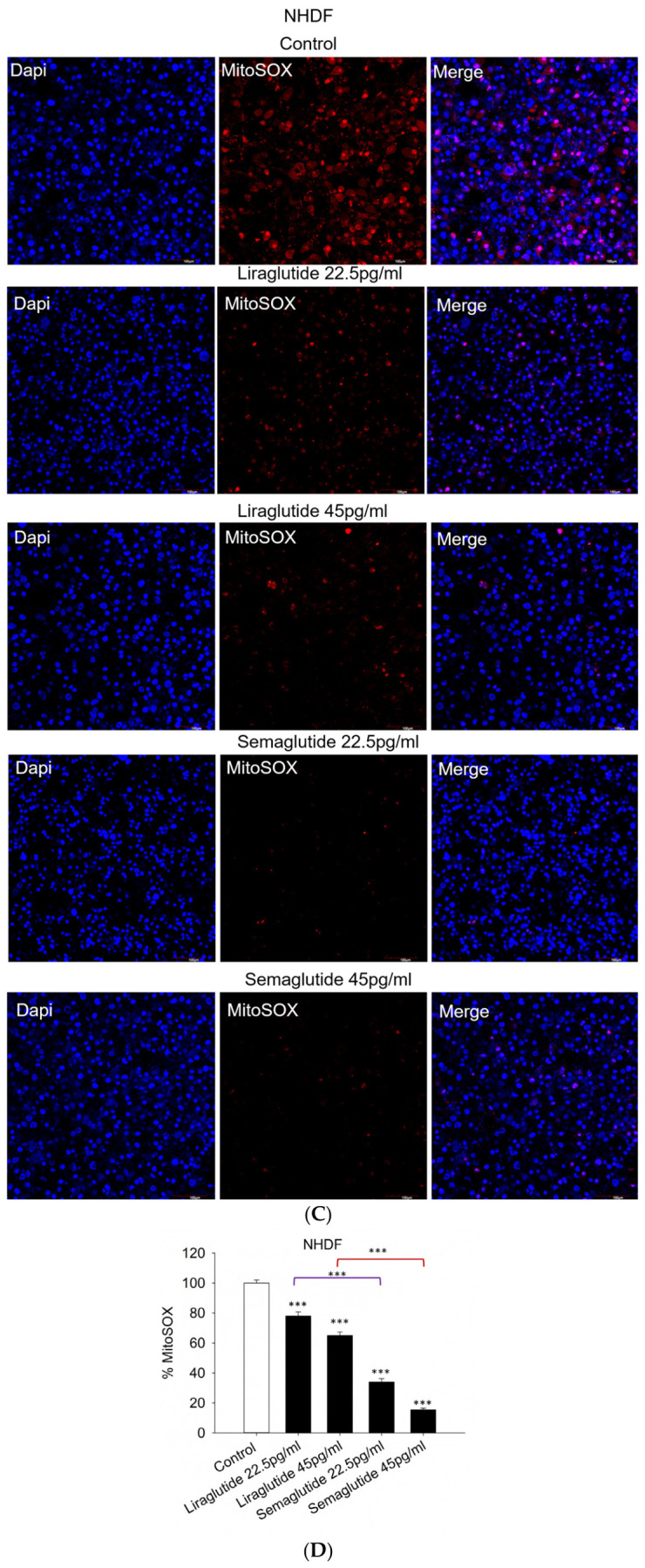
Evaluation of intracellular ROS levels and mitochondrial superoxide production. (**A**). NHDF cells were exposed to a diabetic-like environment and treated with liraglutide or semaglutide (22.5 and 45 pg/mL) for 48 h. The **left** panel shows nuclear DAPI staining (blue), the middle panel shows ROS-specific fluorescence (green), and the **right** panel shows merged images of ROS and DAPI. Images were acquired at 20× magnification, with a scale bar of 100 μm. (**B**). The bar graphs present the quantitative analysis of ROS levels, expressed as mean ± SD from three independent experiments (N = 3). Statistical analysis was performed using one-way ANOVA followed by Dunnett’s T3 post hoc test. Significance is indicated as * *p* < 0.05 and *** *p* < 0.001 versus the control group. Comparisons between the two treatment groups were performed using an independent-samples *t*-test, with *** *p* < 0.001 indicating statistical significance. (**C**). Mitochondrial superoxide production was assessed using MitoSOX™. NHDF cells were exposed to a diabetic-like environment and treated with liraglutide or semaglutide (22.5 and 45 pg/mL) for 48 h. The **left** panel shows nuclear DAPI staining (blue), the middle panel shows MitoSOX™-specific fluorescence (red) indicating mitochondrial superoxide, and the **right** panel shows merged images of MitoSOX™ and DAPI. Images were acquired at 20× magnification, with a scale bar of 100 μm. (**D**). The bar graphs show the quantitative analysis of mitochondrial superoxide levels, expressed as mean ± SD from three independent experiments (N = 3). Statistical significance was determined using one-way ANOVA followed by Dunnett’s T3 post hoc test. Significance levels are indicated as *** *p* < 0.001, compared to the control group. Comparisons between the two drug treatment groups were performed using an independent-samples *t*-test, with significance indicated as *** *p* < 0.001. ANOVA, analysis of variance; Dapi, 4′,6-diamidino-2-phenylindole; DCFDA, 2′,7′-dichlorofuorescein diacetate; NHDF, normal human dermal fibroblasts; MitoSOX, mitochondrial superoxide indicator; ROS, reactive oxygen species; SD, standard deviation.

To further investigate mitochondrial apoptotic signaling under diabetic conditions and after treatment with liraglutide or semaglutide, intracellular ROS generation was quantified using the DCFH-DA fluorescent probe, and mitochondrial superoxide levels were measured with MitoSOX. As shown in Figure 4A,B, both liraglutide and semaglutide at all tested concentrations significantly reduced ROS production in NHDF cells compared with the control group. Notably, semaglutide at 22.5 and 45 pg/mL produced a greater reduction in intracellular ROS levels than liraglutide at the corresponding concentrations in inter-drug comparisons. Moreover, assessment of mitochondrial superoxide showed that liraglutide (22.5 and 45 pg/mL) and semaglutide (22.5 and 45 pg/mL) significantly decreased mitochondrial superoxide levels relative to the control. Notably, semaglutide at 22.5 and 45 pg/mL induced a larger decrease in mitochondrial superoxide levels than liraglutide at the corresponding concentrations in inter-drug comparisons (Figure 4C,D).

### 2.5. AGEs and RAGE Expression

**Figure 5:**
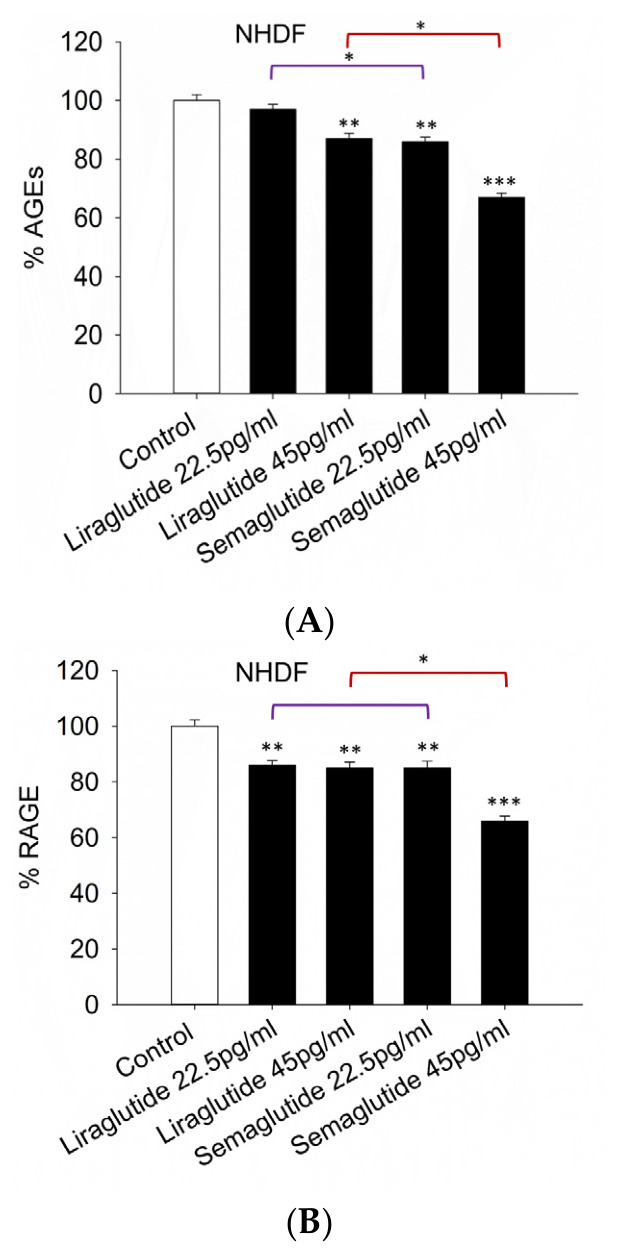
Concentration of AGEs and RAGE in cell culture supernatants. (**A**,**B**). Intracellular levels of AGEs and RAGE concentrations were measured using a colorimetric assay kit. NHDF cells were exposed to a diabetic environment and treated with liraglutide or semaglutide (22.5 and 45 pg/mL) for 48 h. The data are expressed as mean ± SD from three independent experiments (N = 3). Statistical significance was assessed using one-way ANOVA followed by Dunnett’s T3 post hoc test. Significance levels are indicated as ** *p* < 0.01 and *** *p* < 0.001 when compared to the control group. Comparisons between the two drug treatment groups were performed using an independent-samples *t*-test, with significance indicated as * *p* < 0.05. AGEs, advanced glycation end-products; ANOVA, analysis of variance; NHDF, normal human dermal fibroblasts; RAGE, receptor for AGEs; SD, standard deviation.

The impact of semaglutide on the production of AGEs and RAGE was subsequently investigated. Liraglutide (45 pg/mL) and semaglutide (22.5 and 45 pg/mL) significantly reduced AGE levels, and all tested concentrations of both agents decreased RAGE expression in NHDF cells in a dose-dependent manner compared with the control group. Importantly, semaglutide at 22.5 and 45 pg/mL induced a greater reduction in AGEs and RAGE expression than liraglutide at the corresponding concentrations in inter-drug comparisons (Figure 5A,B).

### 2.6. Antioxidant Response-Related Genes

**Figure 6:**
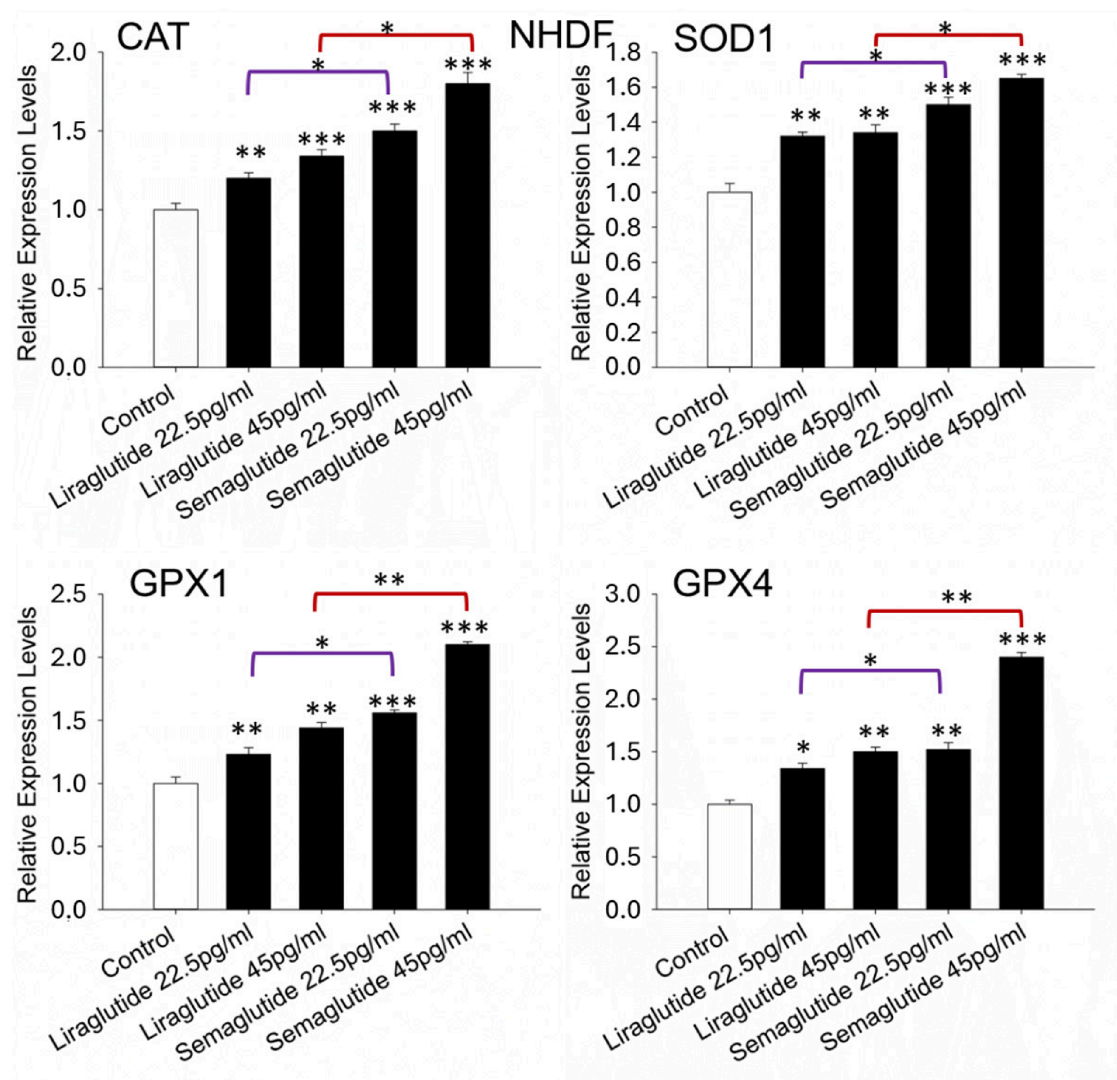
Relative expression levels of *SOD1*, *CAT*, *GPX1*, and *GPX4* in NHDF cells. NHDF cells were exposed to a diabetic-like environment and subsequently treated with liraglutide or semaglutide (22.5 and 45 pg/mL) for 48 h. *ACTB* was used as the internal reference gene. Data are presented as mean ± SD from three independent experiments (N = 3). Statistical analysis was performed using one-way ANOVA followed by Dunnett’s T3 post hoc test. Significance levels are indicated as * *p* < 0.05, ** *p* < 0.01 and *** *p* < 0.001 compared with the control group. Comparisons between the two drug treatment groups were performed using an independent-samples *t*-test, with significance indicated as * *p* < 0.05 and ** *p* < 0.01. ACTB, actin beta; ANOVA, analysis of variance; CAT, catalase; GPX1, glutathione peroxidase; GPX4, glutathione peroxidase-4; NHDF, normal human dermal fibroblasts; SD, standard deviation; SOD1, superoxide dismutase 1.

The effects of liraglutide and semaglutide on the cellular antioxidant response were evaluated by measuring the gene expression levels of superoxide dismutase 1 (*SOD1*), catalase (*CAT*), glutathione peroxidase-1 (*GPX1*), and glutathione peroxidase-4 (*GPX4*) in NHDF cells. All concentrations of liraglutide and semaglutide significantly increased the expression of these genes relative to controls. Moreover, semaglutide at 22.5 and 45 pg/mL induced a greater increase in the expression of these genes than liraglutide at the corresponding concentrations in inter-drug comparisons (Figure 6).

### 2.7. Protein Expression Levels

**Figure 7:**
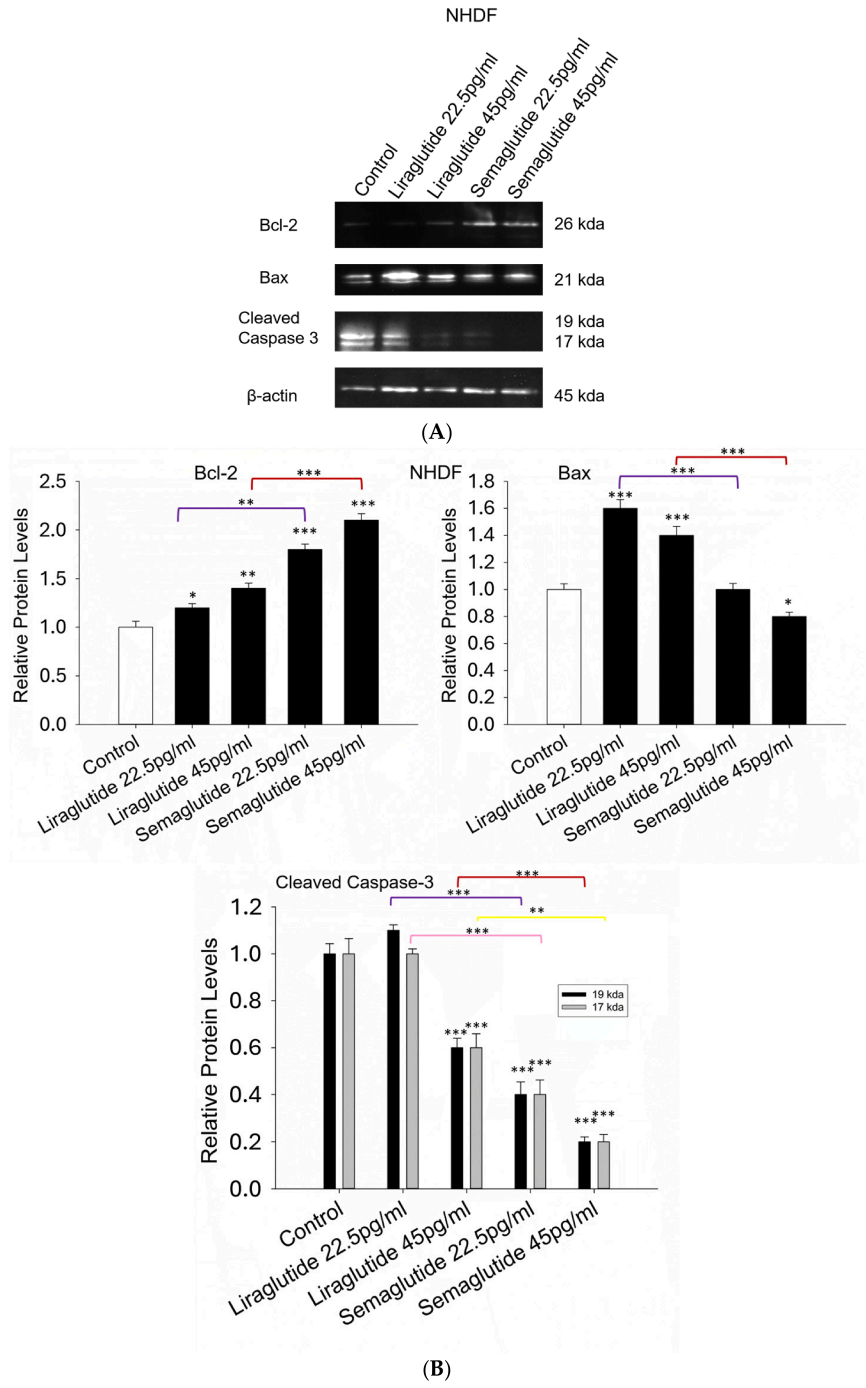
Western blot analysis. (**A**) Western blot analysis of Bcl-2, Bax and Cleaved Caspase-3 on NHDF. B-actin is the loading Control. (**B**) The bar diagram represents the quantitative data. The data is presented as mean ± SD of three independent experiments (N = 3). Statistical significance was determined by one-way ANOVA followed by the Dunnett’s T3 post hoc test; where * *p*-value < 0.05, ** *p*-value < 0.01 and *** *p*-value < 0.001 in comparison with control group. Comparisons between the two drug treatment groups were performed using an independent-samples *t*-test, with significance indicated as ** *p* < 0.01 and *** *p* < 0.001. ANOVA, analysis of variance; Bcl-2, B-cell lymphoma 2; SD, standard deviation.

To evaluate apoptosis, apoptosis-related proteins were quantified in NHDFs exposed to a diabetic environment and subsequently treated with liraglutide or semaglutide. Both treatments increased the anti-apoptotic protein Bcl-2. A significant reduction in the pro-apoptotic protein Bax was observed only following treatment with semaglutide at 45 pg/mL. In contrast, liraglutide treatment resulted in increased Bax expression at both tested concentrations compared with the control group. Importantly, liraglutide at 45 pg/mL and semaglutide at 22.5 and 45 pg/mL significantly decreased the levels of cleaved caspase-3 compared with the control group. Moreover, semaglutide at 22.5 and 45 pg/mL induced a greater increase in Bcl-2 expression than liraglutide at the corresponding concentrations in inter-drug comparisons. Semaglutide at 22.5 and 45 pg/mL also produced a more pronounced decrease in Bax than liraglutide at the corresponding concentrations, and a stronger reduction in cleaved caspase-3 in inter-drug comparisons (Figure 7A,B). B-actin expression was assessed across all groups and showed no significant differences, supporting its use as a stable loading control in this experiment.

### 2.8. Semaglutide Increased Wound Closure Capacity on NHDF

**Figure 8:**
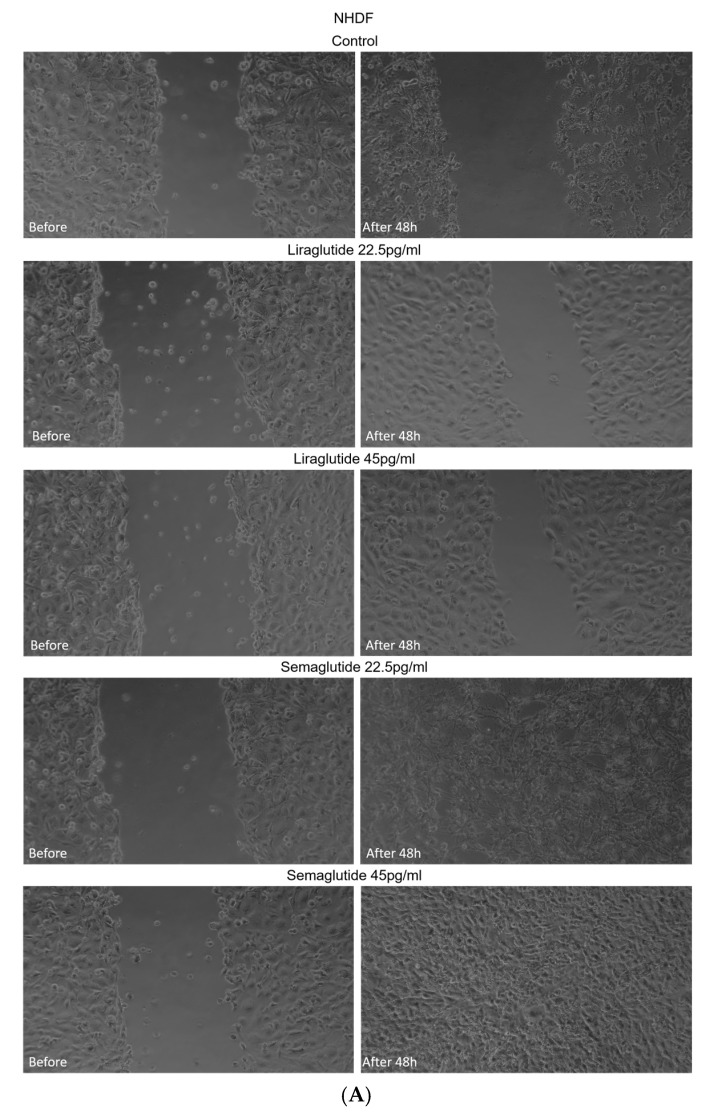

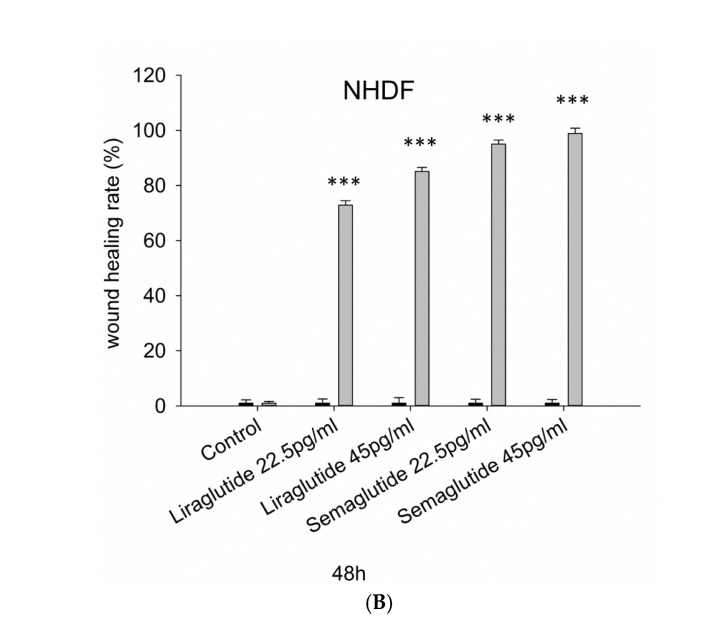
% NHDF wound healing rate. NHDFs were cultured in a diabetic environment and subjected to a scratch wound assay. Cells were treated with liraglutide or semaglutide at 22.5 or 45 pg/mL for 48 h. (**A**). Representative images (5× magnification) show wound width at time 0 (indicated by black arrows) and wound closure after 48 h (indicated by gray arrows). Wound closure was quantified using ImageJ. (**B**). Results show that liraglutide and semaglutide significantly accelerated wound healing relative to baseline (0 h). Data are expressed as mean ± SD from three independent experiments (N = 3). Statistical significance was determined by one-way ANOVA followed by Dunnett’s T3 post hoc test, with significance levels indicated as *** *p* < 0.001 compared to baseline (0 h). ANOVA, analysis of variance; NHDF, normal human dermal fibroblasts; SD, standard deviation.

The increase in NHDF survival and proliferation following liraglutide or semaglutide treatment under diabetic conditions prompted assessment of wound-healing capacity. A scratch wound assay was performed on NHDFs and wound closure was monitored for 48 h. Wound area was quantified at 48 h to evaluate treatment effects. Both liraglutide and semaglutide significantly enhanced wound closure relative to baseline, with semaglutide leading to almost complete closure by 48 h (Figure 8A,B).

### 2.9. Wound Healing Evaluation-Related Genes on NHDF

The relative expression levels of genes associated with different stages of wound healing were evaluated to assess wound closure. The inflammatory phase was examined by measuring the expression of interleukin-1 beta (*IL1B*) and interleukin-2 (IL2). Epidermal growth factor (*EGF*) expression was analyzed to evaluate cellular proliferation. For extracellular matrix (ECM) remodeling, the expression levels of collagen type I alpha 1 chain (*COL1A1*), collagen type III alpha 1 chain (*COL3A1*), collagen type IV alpha 1 chain (COL4A1), collagen type VI alpha 1 chain (*COL6A1*), and matrix metallopeptidase 3 (*MMP3*) were measured.

**Figure 9:**
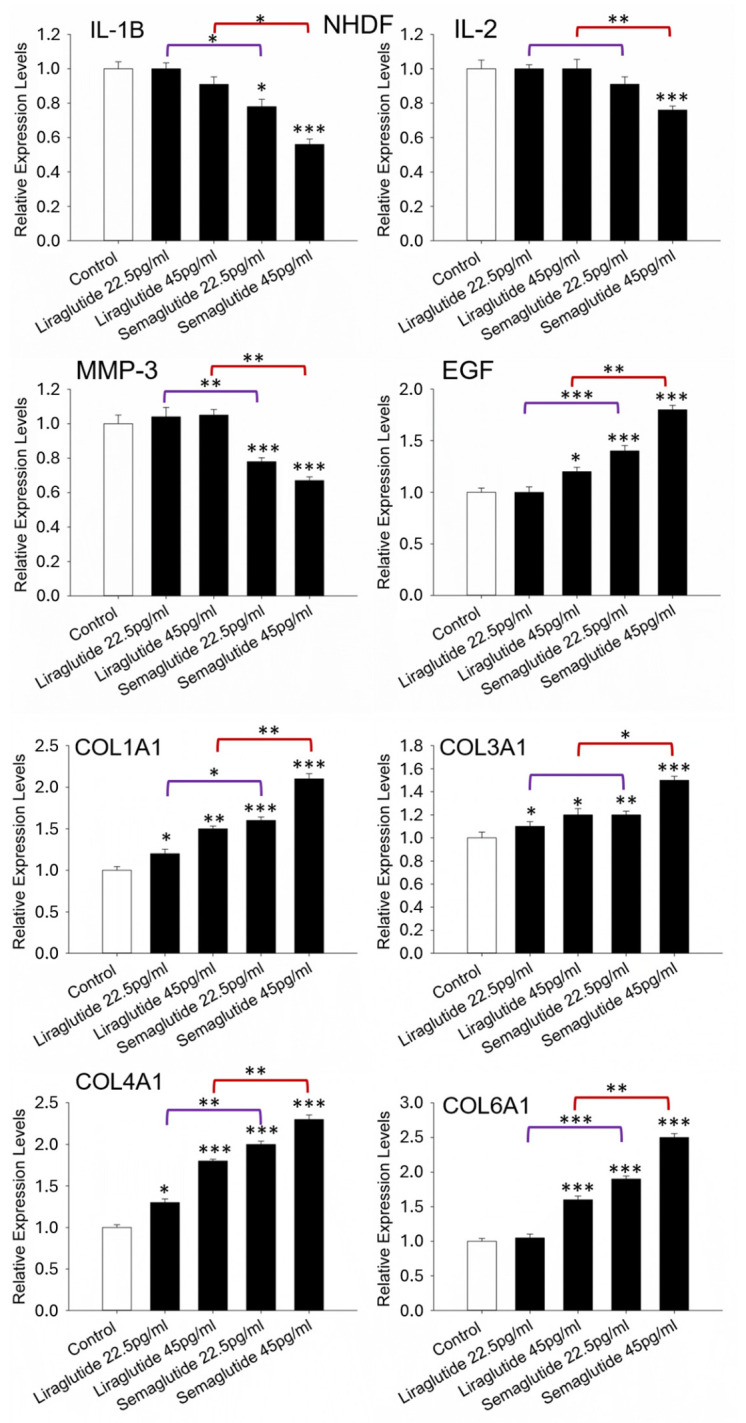
Relative expression levels of *IL1B*, *IL2*, *MMP3*, *EGF*, *COL1A1*, *COL3A1*, *COL4A1*, and *COL6A1* in NHDF cells. NHDFs were exposed to a diabetic environment, subjected to a scratch wound assay, and subsequently treated with liraglutide or semaglutide (22.5 and 45 pg/mL) for 48 h. *ACTB* was used as the internal reference gene. Data are presented as mean ± SD from three independent experiments (N = 3). Statistical analysis was performed using one-way ANOVA followed by Dunnett’s T3 post hoc test. Significance levels are denoted as * *p* < 0.05, ** *p* < 0.01 and *** *p* < 0.001 compared to the control group. Comparisons between the two drug treatment groups were performed using an independent-samples *t*-test, with significance indicated as * *p* < 0.05, ** *p* < 0.01, and *** *p* < 0.001. ACTB, actin beta; ANOVA, analysis of variance; COL1A1, collagen type I alpha 1 chain; COL3A1, collagen type III alpha 1 chain; COL4A1, collagen type IV alpha 1 chain; COL6A1, collagen type VI alpha 1 chain; EGF, epidermal growth factor; IL1B, interleukin 1 beta; IL2, interleukin 2; MMP3, matrix metallopeptidase 3; NHDF, normal human dermal fibroblasts; SD, standard deviation.

Compared with the control group and in inter-drug comparisons, semaglutide significantly downregulated IL1B, IL2, and MMP3, consistent with reduced inflammation and matrix degradation. Compared with the control group, liraglutide (45 pg/mL) and semaglutide (22.5 and 45 pg/mL) significantly upregulated EGF and COL6A1, while all tested concentrations of both drugs markedly increased COL1A1, COL3A1, and COL4A1 expression. In inter-drug comparisons, semaglutide at 22.5 and 45 pg/mL induced greater upregulation of EGF and all collagen genes than liraglutide at the corresponding concentrations. Together, these findings suggest that liraglutide and semaglutide promote wound healing by suppressing inflammation, stimulating proliferation, and enhancing collagen synthesis and extracellular matrix remodeling, with semaglutide exerting a more pronounced effect (Figure 9).

## 3. Discussion

GLP-1R protein levels were measured by ELISA in cell lysates from NHDFs and NHEKs at passages 2–5. GLP-1R was detected in NHDFs at 1.21 ± 0.0019 ng/mL, whereas no detectable signal was observed in NHEKs, indicating cell type-specific expression under the experimental conditions (Supplementary Figure S1). In this study, we examined the cytoprotective and proregenerative effects of liraglutide and semaglutide on NHDF and NHEK cells under diabetic-like conditions. To mimic the diabetic microenvironment, hyperglycemia and oxidative stress were combined, two major factors contributing to impaired wound healing in DM, as extensively described in previous studies [5,15,16]. Our results showed that both liraglutide and semaglutide had protective effects on NHDFs, and semaglutide had a greater effect. In contrast, neither GLP-1 RA showed protective effects on NHEK cells. In particular, semaglutide significantly improved cell viability and proliferation in NHDFs under diabetic-like conditions, whereas liraglutide also showed some protective effects, albeit to a lesser extent. These findings support the data on the cytoprotective and antioxidant properties of GLP-1 RA and further extend their importance to the survival of NHDF cells under diabetic conditions [17].

Additionally, we demonstrated a significant upregulation of the cell proliferation marker Ki-67 and the satellite cell marker Pax-7 in response to semaglutide, an effect not observed with liraglutide. Although Pax-7 is conventionally recognized as a canonical marker for skeletal muscle satellite cells, cell lineage studies have demonstrated that Pax-7-expressing progeny act as a source of dermal cells during the repair process and actively contribute to skin wound healing [18]. This suggests that semaglutide may be more effective in promoting fibroblast proliferation and regeneration, which is in line with its better performance in the wound scratch assay. Semaglutide-treated NHDFs almost completely closed their wounds within 48 h, while liraglutide-treated NHDFs only partially closed their wounds. This indicates that semaglutide might have a clinical benefit in the acceleration of wound healing, which is important in the cessation of chronic DFUs [4,19]

Importantly, both liraglutide and semaglutide decreased apoptosis in NHDFs. Importantly, the decrease in pro-apoptotic markers with semaglutide was more pronounced, especially at higher concentrations, highlighting its augmented anti-apoptotic potential. Semaglutide-induced reduction in Bax suggests a potential shift toward a more anti-apoptotic or cell-survival-favoring profile under these conditions. In contrast, the increase in Bax expression observed with liraglutide at both concentrations indicates a more complex or differential modulation of apoptosis-related pathways, which may reflect distinct downstream signaling effects between the two GLP-1 receptor agonists. This is of great importance since excessive fibroblast apoptosis impedes tissue repair in diabetic wounds [5,20]. Bax and cleaved caspase-3 reflect different stages of apoptosis and may not change in parallel. Liraglutide may have reduced terminal apoptosis while only partially affecting upstream mitochondrial stress signaling under diabetic conditions.

Although GLP-1 RAs did not exert detectable effects on NHEK function in our in vitro system, the pronounced responses in NHDFs still support a potentially beneficial impact on wound healing in vivo [21]. Dermal fibroblasts are key regulators of all phases of repair through extracellular matrix deposition, wound contraction and paracrine signaling with immune and vascular cells, whereas keratinocytes mainly drive re-epithelialization. The strong cytoprotective, antioxidant, anti-apoptotic and pro-regenerative effects of semaglutide, and to a lesser extent liraglutide, on NHDFs therefore suggest that GLP-1 RAs may primarily enhance fibroblast-mediated aspects of healing, which could improve overall wound closure even if their direct actions on keratinocytes are limited. At the same time, our monolayer in vitro model cannot reproduce the full complexity of in vivo wound repair, and these findings should be considered hypothesis-generating; dedicated animal studies and clinical trials are required to determine whether the fibroblast-targeted effects we observed translate into clinically meaningful benefits for wound healing [21].

Oxidative stress is a major driver of diabetic complications and impaired wound healing [5]. Both liraglutide and semaglutide significantly decreased intracellular ROS and mitochondrial superoxide, with semaglutide producing a greater reduction Both agents also upregulated antioxidant enzyme gene expression (*SOD1*, *CAT*, *GPX1*, *GPX4*) consistent with previous animal and clinical reports of GLP-1 RA antioxidant activity [8], an effect that likely is crucial for protecting skin cells from persistent oxidative injury in DM [22]. Moreover, both treatments reduced AGE and RAGE expression which are key mediators of chronic inflammation and tissue damage in DM with semaglutide showing the stronger effect, further supporting its superior cytoprotective profile.

Additionally, we conducted gene expression analysis exclusively in NHDF cells to further extend our findings. The analysis showed that semaglutide is able to modulate important pathways in wound healing. Both GLP-1 RAs induced upregulation of collagen genes (*COL1A1*, *COL3A1*, *COL4A1*, *COL6A1*) that are important for ECM synthesis and tissue remodeling [17]. In the meantime, semaglutide suppressed more effectively pro-inflammatory cytokines (*IL1B*, *IL2*) and *MMP3*, which are generally increased in chronic wounds and lead to excessive matrix degradation and delayed healing [5]. The increase in *EGF* by both drugs is indicative of increased proliferative and migratory ability thereby facilitating wound repair.

Collectively, these findings establish semaglutide as a potent agent for reducing oxidative stress, apoptosis, and inflammation while promoting ECM remodeling and wound closure in human skin cells under diabetic conditions. These effects appear to be more pronounced than those exerted by liraglutide.

Positive effects of GLP-1RA were found only in NHDF and not NHEK it is well established that fibroblasts are mainly involved in all phases of wound healing. The clinical implications are considerable, as improved wound healing could reduce the incidence of DFUs and related amputations major sources of morbidity in DM [4,19,23]. Research indicates that semaglutide exhibits approximately 3-fold greater potency at GLP-1 receptors compared to liraglutide. This higher potency is due to structural differences that enhance its receptor-binding affinity and result in superior glycemic effects and weight loss [24].

Our study, however, has some limitations. Liraglutide and semaglutide are not administered at equivalent doses in clinical practice; liraglutide is typically given as a once-daily subcutaneous injection (up to 1.8 mg/day for type 2 diabetes), whereas semaglutide is administered once weekly (up to 1 mg/week for type 2 diabetes or 2.4 mg/week for obesity), reflecting important differences in pharmacokinetics and receptor potency [25];

Because our model uses normal donor cells exposed to an acute metabolic challenge, it does not fully reflect the chronic and multifactorial biology of diabetic foot ulcers; therefore, the translational relevance of these findings should be interpreted with caution. In addition, these experiments were performed in vitro and thus cannot fully recapitulate the complexity of wound healing in vivo, where multiple cell types and systemic factors interact. Accordingly, further validation in animal models and clinical studies will be necessary to confirm the translational potential of these findings.

## 4. Material and Methods

### 4.1. Cell Culture and GLP-1R Measurements

Normal human dermal fibroblasts (NHDFs) derived from adult human skin were obtained from Lonza Clonetics™ (Catalog No. CC-2511, Lonza Walkersville, USA). NHDFs were maintained according to the manufacturer’s instructions in Fibroblast Growth Basal Medium (FGM™; Catalog No. CC-3131, Lonza Walkersville, USA). Normal human epidermal keratinocytes (NHEKs) derived from healthy adult human skin were obtained from Lonza Clonetics™ (Catalog No. 00192627, Lonza Walkersville, USA). NHEKs were maintained according to the manufacturer’s instructions in Gold Keratinocyte Growth Medium BulletKit (KGM; Catalog No. 00192060, Lonza Walkersville, USA). Cells were passaged at 70–80% confluence using ReagentPack™ (Catalog No. CC-5034, Lonza Walkersville, USA) for subculture, and all experiments were performed using cells between passages 2 and 5.

Both NHDF and NHEK cells were pre-incubated with 30 mM glucose and 100 μM H_2_O_2_ for 12 h to simulate a diabetic environment [15,21,26]. After this pre-incubation, cells were washed with 1× Phosphate-Buffered Saline (PBS; Catalog No. L0615-1000, Biowest, Business Park Ln, Riverside, MO, USA) and then cultured for 48 h in standard growth medium supplemented with liraglutide or semaglutide (22.5 or 45 pg/mL). Control cells were subjected to the same diabetes-mimicking pre-incubation and vehicle exposure (0.1% DMSO) but did not receive GLP-1 receptor agonists. For the assays, cells were inoculated at a density of 6.0 × 10^3^ cells per well in 96-well plates, 2.5 × 10^5^ cells per well in 24-well plates and eight-chamber slides, and 1.0 × 10^6^ cells per well in 6-well plates. The concentrations of semaglutide and liraglutide utilized in this study were derived from those documented in a previous study [27,28].

Liraglutide (≥95% purity, code 204656-20-2, Cayman Chemical, Ann Arbor, MI, USA) and Semaglutide (≥95% purity, code 29969, Cayman Chemical, USA) were solubilized in 1 mg/mL DMSO (catalog no. 25300-062, Thermo Scientific, Fremont, CA, USA) owing to their restricted solubility in water. The DMSO concentration in the culture medium was sustained at 0.1% *v*/*v*. A 30% H_2_O_2_ stock solution (catalog no. 822287, Sigma-Aldrich, USA) was produced and subsequently diluted in the culture medium to achieve a final concentration of 100 μM. GLP-1R protein levels were measured in lysates from NHDFs and NHEKs at passages 2–5 using the Human GLP-1R (glucagon-like peptide 1 receptor) ELISA Kit (Catalogue No. EH1151, FineTest, China) according to the manufacturer’s instructions.

All cell culture supplies were obtained from Falcon (VWR International Eurolab). All other chemicals used in this study were of analytical grade and purchased from reputable commercial suppliers.

### 4.2. MTT Measurement

NHDF and NHEK cells were inoculated onto 96-well plates with six biological replicates, adhering to the previously outlined protocol. Upon conclusion of the treatment period, MTT reagent (#30006, Biotium Inc., Fremont, CA, USA) was introduced to each well, and the assay was performed in accordance with the prescribed methodology [29,30].

### 4.3. Detection of Cell Apoptosis

NHDF and NHEK cells were cultured in triplicate within 6-well plates, and apoptosis levels were assessed via propidium iodide (PI) staining, in accordance with the previously outlined protocol [29,31]. Cells were fixed and stained with propidium iodide, and DNA content was analyzed by flow cytometry; apoptotic cells were quantified as the sub-G_0_/G_1_ population in the PI histogram. Measurements were performed in three biological replicates.

### 4.4. Detection of Ki-67 and Pax-7 Co-Expression via Immunofluorescence

A recombinant anti-Ki67 antibody (1:200, Abcam, ab16667) and Pax-7 (1:100, sc-81648, Santa Cruz Biotechnology) were used according to the manufacturer’s instructions. Cells were fixed in 4% paraformaldehyde in PBS for 10–15 min at room temperature, washed three times with PBS, and permeabilized with 0.25% Triton X-100 in PBS for 10 min. After three PBS washes, non-specific binding was blocked for 30 min at room temperature in PBS containing 0.1% BSA. Primary antibodies were diluted in PBS/0.1% BSA and incubated overnight at 4 °C. The following day, coverslips were washed three times with PBS and incubated for 45–60 min at room temperature with Atto-594-conjugated donkey anti-rabbit (Thermo Fisher, A-21207; 1:500) for Ki-67 and Atto-488-conjugated donkey anti-mouse (Thermo Fisher, A-21202; 1:500) for Pax-7. After secondary antibody incubation, samples were washed three times with PBS. Nuclei were counterstained with DAPI (4′,6-diamidino-2-phenylindole dihydrochloride; Molecular Probes) at 0.1 µg/mL in 0.9% NaCl for 5–10 min, washed once with PBS, and mounted with antifade medium. Imaging was performed on a confocal microscope (Olympus FluoView FV1000).

### 4.5. Detection of Intracellular Reactive Oxygen Species Level

Intracellular ROS levels were assessed using the DCFDA Cellular ROS Detection Kit (ab113851, Abcam) according to the manufacturer’s instructions. NHDFs were cultured on 8-chamber slides as described above and incubated with 25 μM DCFDA in phenol red-free DMEM supplemented with 15% FBS for 45 min at 37 °C in a humidified incubator. Following staining, cells were washed twice with PBS, and nuclei were counterstained with DAPI (1:1000 in PBS; D9542, Sigma-Aldrich). ROS-dependent green fluorescence was then visualized using a confocal microscope (Olympus FluoView FV1000; excitation, 488 nm; emission, 525 nm). Fluorescence intensity was quantified using ImageJ software (https://imagej.nih.gov/ij/, accessed on 10 January 2026), with at least three microscopic fields analyzed per treatment condition.

### 4.6. Detection of Mitochondrial Superoxide

Mitochondrial superoxide levels were evaluated using the MitoSOX™ Red mitochondrial superoxide indicator (M36008, Thermo Fisher/Molecular Probes) for live-cell imaging. NHDFs were plated on 8-chamber slides and exposed to liraglutide or semaglutide for 48 h (three biological replicates per condition). Following treatment, cells were incubated with MitoSOX™ Red according to the manufacturer’s recommendations, rinsed with PBS, and examined by confocal microscopy (Olympus FluoView FV1000; excitation 510 nm, emission 580 nm). Nuclei were counterstained with DAPI (1:1000 in PBS; D9542, Sigma-Aldrich). For each treatment, at least three microscopic fields were acquired, and MitoSOX™ fluorescence intensity was quantified using ImageJ to estimate relative mitochondrial superoxide production.

### 4.7. AGEs and RAGE Measurements

The concentrations of AGEs in cell culture supernatants and RAGE in cell pellets were quantified using commercial ELISA kits (human AGE, EH0622; human RAGE, EH0408; Fine Test, China) according to the manufacturer’s instructions. Cells were seeded at 8000 per well in 24-well plates (three replicates per treatment) and treated with liraglutide or semaglutide for 48 h. After treatment, cells were collected and homogenized in 500 μL assay buffer on ice for 1 h, then centrifuged at 10,000 rpm for 10 min to remove insoluble debris. The resulting supernatants were analyzed by ELISA, and standard curves within the manufacturer’s specified range were used to calculate AGE and RAGE concentrations. Absorbance was measured at 450 nm with a Multiskan™ FC Microplate Photometer (Thermo Scientific, USA).

### 4.8. RNA Preparation and Quantitative Real-Time PCR

Five hundred nanograms of total RNA was extracted from cells seeded in 6-well plates using the NucleoSpin RNA Kit (Macherey-Nagel, Düren, Germany) according to the manufacturer’s instructions. The integrity of 1 μL of the eluted RNA was assessed by 1% agarose gel electrophoresis, and RNA concentration was determined spectrophotometrically at 260 nm using a NanoDrop spectrophotometer. Complementary DNA (cDNA) was synthesized using the PrimeScript RT Reagent Kit (Takara Bio, Japan). Quantitative real-time PCR (RT-qPCR) was performed on a StepOnePlus™ Real-Time PCR System (Applied Biosystems™, Waltham, MA, USA) according to the manufacturer’s instructions. SYBR Green chemistry (1708880, Bio-Rad, Hercules, CA, USA) was used for detection. Primer sequences and gene details are provided in Supplementary Table S1, and primers were designed as previously described [32]. Relative gene expression levels were calculated using the 2^−ΔΔCt^ method [32,33] with actin beta (ACTB) serving as the reference gene, as its expression is unaffected by the experimental treatments [34]. Data analysis was conducted with three biological replicates.

### 4.9. Western Blot Analysis

NHDF cells were seeded in dishes 100 mm as described above [29]. Trypsin/EDTA was subsequently employed for the rapid harvesting of the cells. Following an extensive wash of the cells with PBS, they were centrifuged for 10 min at 4 °C at 200× *g*. The cells were subsequently lysed in an ice-cold RIPA lysis solution for one hour. This buffer comprised 50 mM Tris-HCl at pH 7.4, 150 mM NaCl, 0.1% SDS, 1 mM EDTA, 1% Triton X-100, 1 mM phenylmethylsulfonyl fluoride, a 1:100 protease inhibitor, a 1:100 phosphatase inhibitor, and an additional 1 mM phenylmethylsulfonyl fluoride. Subsequent to the collection of lysates, they were subjected to centrifugation for 10 min at 4 °C at 20,000× *g*. The Bradford protein assay was employed to ascertain the total protein concentrations subsequent to the transfer of the supernatants to a fresh tube. Cell Signaling Technology (Danvers, MA, USA) supplies all antibodies, biotinylated protein ladder (7727, dilution 1:1000), nitrocellulose membranes (12369), 20X lumiGLO reagent, and 20X peroxide (95538S). For Western blotting, equivalent quantities of protein (40 μg) and ladder were resolved using a gradient 7.5–12% sodium dodecyl sulfate polyacrylamide gel and subsequently transferred to nitrocellulose membranes. The immunoblot membranes were treated for 1 h at room temperature in a blocking solution of 5% milk or bovine serum albumin in Tris-Buffered Saline with 0.1% Tween, followed by overnight incubation at 4 °C on a shaker with the primary antibodies. On the next day, the primary antibodies were eliminated, and the membranes were treated with secondary antibodies (anti-rabbit IgG and anti-mouse IgG) at 37 °C for one hour (loading controls, primary and secondary antibodies are specified in Supplementary Table S2). Antibody-binding bands were detected using 20X lumiGLO reagent and 20X Peroxide (dilution 1:20). Bands were detected by chemiluminescence and captured using a digital imaging system. The band density was evaluated using Image Pro Plus version 6.0 software (Media Cybernetics, Rockville, MD, USA).

### 4.10. In Vitro Wound Scratch Assay

A wound-healing scratch assay was used to assess the effects of semaglutide on NHDF cells. NHDFs were seeded into 24-well plates and cultured at 37 °C in a humidified incubator with 5% CO_2_ until a confluent monolayer was formed. A linear scratch was then created with a sterile P200 pipette tip, and detached cells were removed by rinsing with 1× PBS. The wound area was imaged immediately (0 h) using phase-contrast microscopy at 5× magnification, after which cells were treated with liraglutide or semaglutide for 48 h. Wound healing rate (%) was calculated as the percentage reduction in the initial wound area using the formula [(A_0_ − A_t_)/A_0_] × 100, where A_0_ is the wound area at 0 h and A_t_ is the wound area at 48 h [35]. In all scratch-wound experiments, the same wound region was imaged at 0 h and 48 h by using reference marks on the underside of the plate and maintaining identical plate orientation and microscope stage coordinates. Wound area was quantified in ImageJ relative to the 0-h image, and the degree of healing was expressed as the percentage reduction in the initial wound area [36]. All experiments were conducted with three independent biological replicates.

### 4.11. Graph Presentation and Statistical Analysis

All data are presented as mean ± standard deviation (SD). Graphs were generated using SigmaPlot 12.2 (Systat Software Inc., San Jose, CA, USA), and statistical analyses were performed using IBM SPSS Statistics for Windows, Version 22.0 (IBM Corp., Armonk, NY, USA). Differences among multiple groups were assessed using one-way analysis of variance (ANOVA) followed by Dunnett’s T3 post hoc test. Comparisons between the two treatment groups were performed using an independent-samples *t*-test. A *p*-value < 0.05 was considered statistically significant.

## 5. Conclusions

In conclusion, semaglutide exhibits greater antioxidant, anti-apoptotic, anti-inflammatory, and pro-regenerative effects than liraglutide in human fibroblast cells under diabetic conditions. These data support the use of GLP-1 RA and especially semaglutide as promising therapeutic candidates to enhance wound healing and treat diabetic complications and warrant further preclinical and clinical studies.

## Data Availability

The original contributions presented in this study are included in the article. Further inquiries can be directed to the corresponding authors.

## Supplementary Materials

The following supporting information can be downloaded at: https://www.mdpi.com/article/10.3390/ijms27156981/s1.

## Abbreviations

ACTB	Actin beta
AGEs	Advanced glycation end-products
ANOVA	One-way analysis of variance
CAT	Catalase
cDNA	Complementary DNA
COL1A1	Collagen type I alpha 1 chain
COL3A1	Collagen type III alpha 1 chain
COL4A1	Collagen type IV alpha 1 chain
COL6A1	Collagen type VI alpha 1 chain
DAPI	4′,6-diamidino-2-phenylindole dihydrochloride
DFUs	Diabetic foot ulcers
DM	Diabetes mellitus
ECM	Extracellular matrix
EGF	Epidermal growth factor
GLP-1 RAs	Glucagon-like peptide-1 receptor agonists
GLP-1	Glucagon-like peptide-1
GLP-1R	Glucagon-like peptide 1 receptor
GPX1	Glutathione peroxidase-1
GPX4	Glutathione peroxidase-4
H2O2	Hydrogen peroxide
IL1B	Interleukin-1 beta
IL2	Interleukin-2
MMP3	Matrix metallopeptidase 3
NHDF	Normal human dermal fibroblasts
NHEK	Normal human epidermal keratinocytes
PBS	Phosphate-buffered saline
PI	Propidium iodide
RAGE	Receptor for advanced glycation end-products
ROS	Reactive oxygen species
RT-qPCR	Quantitative real-time polymerase chain reaction
SD	Standard deviation
SDS	Sodium dodecyl sulfate
SOD1	Superoxide dismutase 1
T2D	Type 2 diabetes
