# Supplementary material for: Semaglutide and Liraglutide Modulate Oxidative Stress and Wound Repair in Normal Human Skin Cells Exposed to an In Vitro Diabetic-like Environment
Source: Int J Mol Sci. 2026 Aug 3;27(15):6981. doi: 10.3390/ijms27156981 (PMC13467259; doi:10.3390/ijms27156981)
Supplement: Supplementary file 1 [file ijms-27-06981-s001.zip › ijms-4439351-supplementary.pdf]

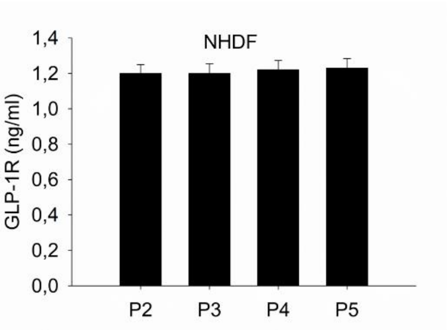

Supplementary Figure S1: Measurement of GLP-1R

GLP-1R levels were measured in cell lysates from NHDF and NHEK; expression was undetectable in NHEK.

A

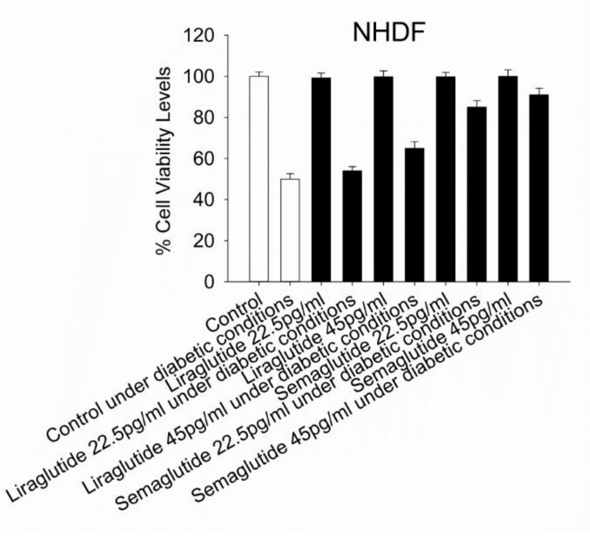

B

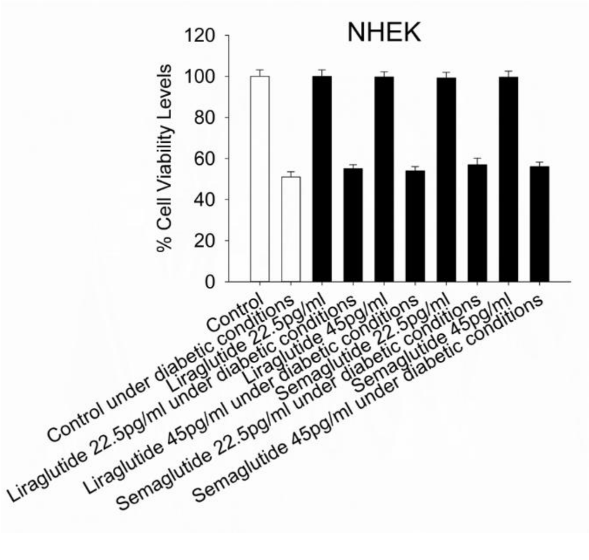

Supplementary Figure S2: Assessment of Cell Viability

A, B. Cell viability of NHDF and NHEK was determined using the MTT assay. Cells were treated with liraglutide or semaglutide (22.5 and 45 pg/ml) for 48 hours. Additionally, NHDF and NHEK cells were exposed to a diabetic environment and treated with liraglutide or semaglutide (22.5 and 45 pg/ml) for 48 hours. The data presented here reflect the outcomes of these treatments.

Supplementary Table S1: Gene name. Accesion No. Primer sequences, Anealinh temperature

Gene Symbol	Gene Name	Accession No	Primer F (5'-3')	Primer R (5'-3')	Annealing Temperature (°C)
ACTB	actin. beta	NM_001101.5	GAGAAGAGCTACGAGCTGCC	AAGGAAGGCTGGAAGAGTGC	60
COL1A1	collagen. type I. alpha 1	NM_000088.4	AGTGGTTTGGATGGTGCCAA	GCACCATCATTTCCACGAGC	60
COL3A1	collagen. type III alpha 1	NM_000090.4	AGCCTGGTAAGAATGGTGCC	CTCCTGGGATGCCATTTGGT	60
COL4A1	collagen. type IV alpha 1	NM_001303110.2	AGGAGTGCCATTGCTTTTTCAA	TGGAAACCAGTCCATGCTCG	60
COL6A1	collagen. type VI alpha 1	NM_001848.3	TTATCAACGACGCCACCGA	TGCGAGTTGCCATCTGAGAAG	60
GPX1	glutathione peroxidase 1	NM_001329455.2	CGATGTTGCCTGGAACTTTGAG	ATGTCAATGGTCTGGAAGCGG	60
GPX4	glutathione peroxidase 4	NM_001845.6	GGAGTTTAGAAGTGCGCCATTC	CCAAAAGCTGTAAGCGTTTGC	60
CAT	catalase	NM_001752.4	ACCAAGGTTTGGCCTCACAA	CCACCCTGATTGTCCTGCAT	60
SOD1	superoxide dismutase 1	NM_000454.5	GGATGAAGAGAGGCATGTTGGA	TAGACACATCGGCCACACCAT	60
Il1B	interleukin 1 beta	NM_000576.3	TTTGAGTCTGCCCAGTTCCC	TTTGGTCCCTCCCAGGAAGA	60
IL2	Interleukin 2 (IL2)	NM_000586.4	GACCCAGGGACTTAATCAGCAAT	GCTGTCTCATCAGCATATTCACAC	60
MMP3	matrix metallopeptidase 3 (MMP3)	NM_002422.5	ACTCCCTGGGTCTCTTTCACT	GAACCGAGTCAGGTCTGTGAG	60
EGF	Epidermal growth factor (EGF)	NM_001178130.3	AACTTCATGGAGGCAGGAGC	CATTACCTGGGGACAGGAGC	60

Supplementary Table S2: List and dilution of antibodies.

**Primary antibodies**	**Dilution**	**Cat**	**RRID**	**Supplier**
Rabbit β-Actin (loading control) polyclonal antibody	1: 2000	#4967	AB_330288	Cell Signaling Technology
Rabbit monoclonal antibody beta cell lymphoma 2 (Bcl-2)	1: 1000	#3498	AB_1903907	Cell Signaling Technology
Rabbit polyclonal antibody Bcl-2 associated X, apoptosis regulator (Bax)	1: 1000	#2772	AB_10695870	Cell Signaling Technology
Rabbit polyclonal antibody caspase-3	1: 1000	#9662	AB_331439	Cell Signaling Technology
**Secondary antibodies**	**Dilution**	**Cat**	**RRID**	**Supplier**
Anti-rabbit IgG horseradish peroxidase-conjugated secondary polyclonal antibody	1: 2000	#7074	AB_2099233	Cell Signaling Technology
